# Chocolate Milk Consequences: A Pilot Study Evaluating the Consequences of Banning Chocolate Milk in School Cafeterias

**DOI:** 10.1371/journal.pone.0091022

**Published:** 2014-04-16

**Authors:** Andrew S. Hanks, David R. Just, Brian Wansink

**Affiliations:** 1 Dyson School of Applied Economics and Management at Cornell University, Ithaca, New York, United States of America; 2 Marketing, Dyson School of Applied Economics and Management at Cornell University, Ithaca, New York, United States of America; University of Missouri-Kansas City, United States of America

## Abstract

**Objectives:**

Currently, 68.3% of the milk available in schools is flavored, with chocolate being the most popular (61.6% of all milk). If chocolate milk is removed from a school cafeteria, what will happen to overall milk selection and consumption?

**Methods:**

In a before-after study in 11 Oregon elementary schools, flavored milk–which will be referred to as chocolate milk–was banned from the cafeteria. Milk sales, school enrollment, and data for daily participation in the National School Lunch Program (NSLP) were compared year to date.

**Results:**

Total daily milk sales declined by 9.9% (p<0.01). Although white milk increased by 161.2 cartons per day (p<0.001), 29.4% of this milk was thrown away. Eliminating chocolate milk was also associated with 6.8% fewer students eating school lunches, and although other factors were also involved, this is consistent with the notion of psychological reactance.

**Conclusions:**

Removing chocolate milk from school cafeterias may reduce calorie and sugar consumption, but it may also lead students to take less milk overall, drink less (waste more) of the white milk they do take, and no longer purchase school lunch. Food service managers need to carefully weigh the costs and benefits of eliminating chocolate milk and should consider alternative options that make white milk more convenient, attractive, and normal to choose.

## Introduction

Chocolate milk can have up to twice as much sugar as white milk and as a result, removing chocolate milk from school cafeterias has been actively debated as a measure to reduce childhood obesity. In response, many school districts have begun to limit or ban the sale of chocolate milk in hopes of reducing students’ total caloric and sugar intake. [1] In contrast, the predominant view of nutrition and medical researchers is that milk has nutrients essential for bone growth and development, [2], [3], [4], [5], [6] leading other school districts to take the position that any milk is better than no milk. [7], [8], [9] What is not known, however, is whether or not changing the availability of chocolate milk would influence other behaviors such as within-meal compensation [10] or after-school snacking patterns. [7], [11], [12], [13] Furthermore, restricting the availability of chocolate milk may lead to adverse economic impacts such as increased milk waste and decreased lunch sales. In general, all flavored milk with added sugar, including chocolate, strawberry, and vanilla, comprises 68.3% of all milk available in schools. [14] Since chocolate milk is the most common flavor (61.6% of all milk and 90.2% of flavored milk [14]) we will refer to all types of flavored milk as chocolate milk.

Of the students who purchase lunches served as part of the National School Lunch Program (NSLP), two-thirds choose chocolate over white milk [15], [16]. Because most students drink chocolate milk for taste rather than nutrition [17], banning chocolate milk might not lead students to immediately substitute over to white milk. Fortunately, making white milk the default choice, and making chocolate milk less convenient without eliminating it, could increase white milk selection immediately, and with little controversy. [18], [19].

## Methods

### Study Design and Data

This pilot study took place in eleven elementary schools within a single Oregon school district. Total population in the district is roughly 157,000 adults and students, and 85% are Caucasian, 7.8% are of Hispanic or Latino origin, and 1.4% are African American. Median household income is $41,326, compared to the national median of $52,762, and 21.5% of the individuals in the district are below the poverty level, compared to the 14.3% national average.

In the 2011–12 school year, school food service in the district removed chocolate milk in grades K-5 and offered skim milk instead. This offers a before versus after evaluation opportunity to explore differences in milk sales after controlling for changes in enrollment between the 2010–2011 school year and the 2011–2012 school year. To assess the potential impact of removing chocolate milk on student behavior, milk sales data were collected during the months of September and October of both school years. September and October were selected because initially the school board had planned to re-introduce chocolate milk after the two-month trial period. Yet, after October, the board decided to continue the policy, though data collection stopped. For the months during the study, the school district provided data for average daily participation rates, overall school enrollment, and percent of students receiving a free or reduced price lunch. Given milk sales data and school enrollment, behavioral changes can be evaluated between the two school years.

Aggregate daily milk waste data were also collected in all but one of the elementary schools during the September and October 2011–2012 school year. After students had completed their meals, they poured their milk, both white and chocolate, into a separate bucket located next to the garbage receptacles. These waste measures were reported as gallons dumped, from which the number of 8-oz cartons wasted can be calculated.

Since milk waste data were not collected in the study schools during the 2010–2011 school year, we compare waste in 2011–2012 to waste measures from 15 other elementary schools from the Midwestern and Eastern United States. From these 15 elementary schools we generated two baseline waste measures. The first baseline measure of milk waste was derived from milk waste data collected during the spring of 2012 in 5 of the 15 elementary schools, which were located in New York State. Additional comparison measures were generated from 10 elementary schools from 10 states in the Midwestern and Eastern United States (not including New York State). These measures were collected during the fall of 2012 when chocolate milk was restricted to the fat free variety.

Milk waste at all 15 schools was collected using the Quarter-Waste Method [20], a reliable visual estimation technique for measuring tray waste in school cafeterias. Waste estimates from this Quarter-Waste Method are highly correlated with standard weighing techniques used to measure waste, with estimated correlation coefficients in excess of 0.90. Moreover, the Quarter-Waste Method generates a mean waste that is within one gram of the amount obtained by weighing milk waste [20].

Removal of chocolate milk was not the only change in this Oregon school district. In the 2010–2011 school year, this school district had a total of 21 elementary schools. Because of enrollment decreases, four of these schools closed prior to the 2011–2012 school year. The remaining students were transferred to other elementary schools in the district, causing wide swings in enrollment (greater than 35% increases) in four of these schools. In addition, two of the schools did not provide data for analysis, thus reducing the number of elementary schools in the analysis to 11 schools.

In addition, there were various changes within the school’s food service. First, at the onset of the 2011–2012 school year, lunch menu cycles changed from a 5-week to a 4-week cycle, reducing the variety of meals, particularly entrées, offered. Parental and student demand, however, led to more frequent offerings of popular entrées such as pizza, tacos, burgers, and breaded chicken products. Bonus items, such as cookies (offered every other Friday), pickles, croutons, cracker products, breadsticks, and whipped topping were also removed. A vegetable side was included with each entrée on a daily basis. In combination with these menu changes the lunch price increased from $2.25 to $2.50 per meal for the full price lunch, most likely decreasing the number of full priced lunches purchased. Notably, cafeterias in the participating elementary schools provided beverage choices of bottle water, milk, and juice to students. No competitive foods (items not sold as part of the National School Lunch Program – NSLP) were available.

### Analysis

In order to better understand the potential impact that removing chocolate milk has on milk sales and intake, the relevant research questions were studied in two phases. First, school level data were used to compare average milk sales between the two school years to determine whether purchases of 1% and fat free milks in the 2011–2012 school year offset the impact on milk sales from eliminating chocolate milk. In each of these comparisons, mixed effects regression methods were used with random effects at the school level. Milk sales for all milk, 1%, milk, fat free milk, and chocolate milk were used as dependent variables in separate regressions. A dummy variable for year captured the impact from the change in milk options and a monthly control was also included in the analysis to account for seasonal variation.

Second, comparisons in milk waste data were studied using an unequal sample size, unequal variance t-test and the degrees of freedom for the test were calculated using the Welch-Satterthwait equation [21]. Since milk waste in the second set of comparison schools was measured differently, an unpaired t-test of the difference in percent milk wasted is an appropriate statistical method. In addition, allowing for unequal variances provides a sensitivity check. Analyses were conducted using Stata 12.

## Results

**Figure 1:**
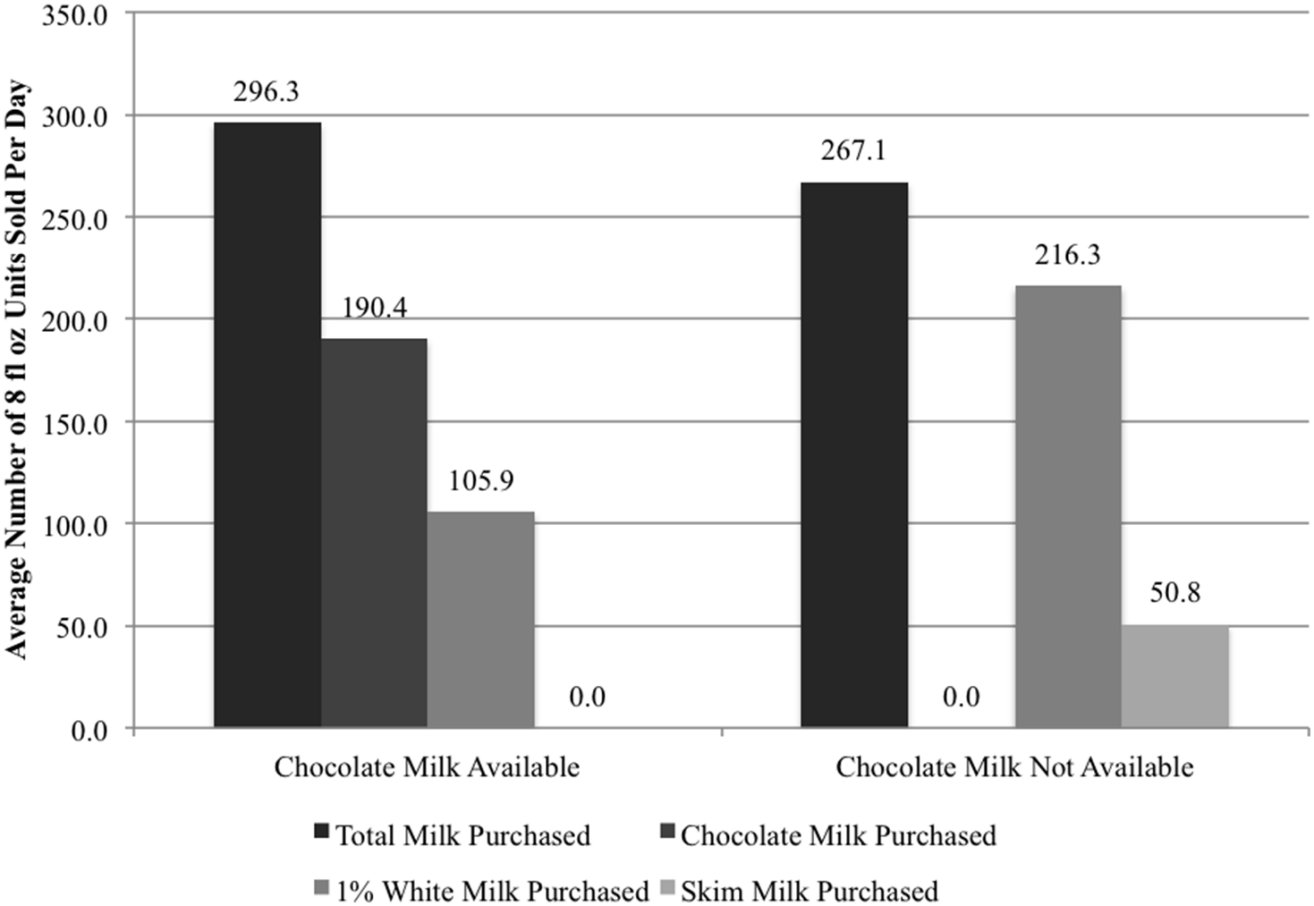
Substituting Skim for Chocolate Milk in 2011 Led to a Decrease in Daily Milk Sales.

As a result of the policy, average sales per school of chocolate milk dropped from 190.4 to 0 (Table 1; Figure 1). Interestingly, 90.1% of chocolate milk sales were recovered by 1% fat and skim white milk options. Indeed, following the elimination of chocolate milk average daily sales per school of white milk increased by 152% between the two school years (from 105.9 to 267.1 cartons; p<0.001; Table 2; Figure 1). This increase suggests that many students were willing to substitute 1% or skim white milk for chocolate milk. Yet, an average of 30 fewer units of milk were sold each day in Sep and Oct of 2011, suggesting that approximately 30 of the 380 students (average enrollment) no longer chose the nutritious beverage. This translates to a 9.9% decrease in milk selection (p<0.01; Table 1).

**Table 1:** Milk Sales Declined When Chocolate Milk Was Banned (standard errors in parentheses).

	Chocolate Milk Available (SY 2010–2011)	Chocolate Milk Not Available (SY 2011–2012)	Difference	% Difference
Average Monthly Total Milk Sales	5334.1	4540.2	−793.9***	−14.90%
(N = 44)	(767.91)	(767.91)	(180.66)	
Average Monthly 1% Milk Sales	1906.8	3677.3	1770.5***	92.90%
(N = 44)	(591.23)	(591.23)	(181.41)	
Average Monthly Chocolate(‘10)/Skim(‘11) Milk Sales	3427.3	863.0	−2564.3***	−74.80%
(N = 44)	(250.37)	(250.37)	(208.23)	
Average Daily Total Milk Sales	296.3	267.1	−29.3**	−9.90%
(N = 44)	(43.91)	(43.91)	(10.31)	
Average Daily 1% Milk Sales	105.9	216.3	110.4***	104.20%
(N = 44)	(34.04)	(34.04)	(11.18)	
Average Daily Chocolate(‘10)/Skim(‘11) Milk Sales	190.4	50.8	−139.6***	−73.30%
(N = 44)	(14.12)	(14.12)	(11.68)	

Results for this table were generate using mixed effects regression methods with random effects at the school level and monthly controls.

*p<0.05.

**p<0.01.

***p<0.001.

**Table 2:** Fewer Students Took Milk When Chocolate Milk Was Banned (standard errors in parentheses).

	Chocolate Milk Available (SY 2010–2011)	Chocolate Milk Not Available (SY 2011–2012)	Difference	% Difference
Average Daily % Students Taking All Milks	77.8%	71.4%	−6.3%**	−8.23%
N = 44	(0.102)	(0.102)	(0.025)	
Average Daily % Students Taking 1%	27.3%	58.4%	31.2%***	113.92%
N = 44	(0.083)	(0.083)	(0.033)	
Average Monthly Chocolate(‘10)/Skim(‘11) Milk Sales	50.5%	12.0%	−37.5%***	−76.24%
N = 44	(0.030)	(0.030)	(0.030)	

Results for this table were generated using mixed effects regression methods with random effects at the school level and monthly controls.

*p<0.05.

**p<0.01.

***p<0.001.

**Figure 2:**
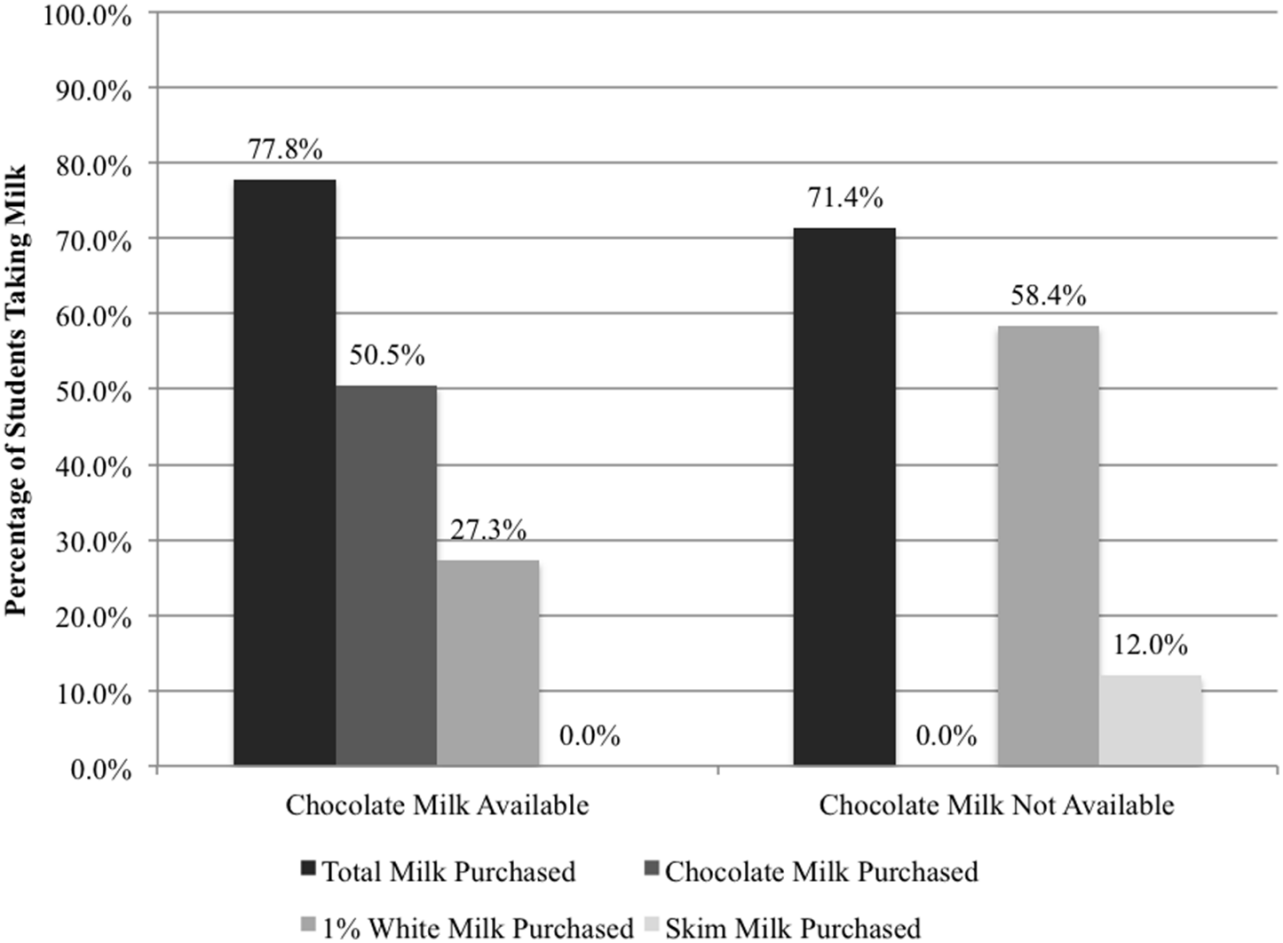
When Chocolate Milk Was Banned the Proportion of Students Taking Milk Declined.

In all schools nation wide, milk is served as a beverage choice for reimbursable meals as well as for purchase a la carte. In terms of total student enrollment (not restricting the student body to NSLP participants), there was an 8.2% decline (p<0.001; Table 2; Figure 2) in the proportion of students who took milk. When chocolate milk was still an option, 77.8% of all students took milk. Once chocolate milk was removed, 71.4% took milk.

When comparing milk types between the school years, there was a 113.9% increase (p<0.001; Table 2; Figure 2) in the percentage of enrolled students taking 1% milk. Moreover, the percentage of students selecting a milk type other than 1% white–including fat free chocolate in the 2010–2011 school year and fat free white in the 2011–2012 school year–decreased by 76.2%. Nutritional benefits to eliminating chocolate milk are an average decrease of 8 grams of sugar and 37 calories in a student’s lunch (not necessarily consumed). These benefits, however, come at a cost of 1 gram of protein, a decrease of 5 percentage points in the daily recommended intake of calcium, and an additional ½ gram of fat per average student’s lunch.

Even though sales data suggest many students willingly substituted chocolate for white milk, sales alone provide no indication whether or not students drank the lower calorie 1% or skim milk on their trays. Using milk waste data, we examine the amount of milk wasted when only white milk was offered. Cafeteria staff collected data on total gallons of milk wasted each day in September and October of the 2010–2011 school year. On average, students wasted 40.9% of the milk they selected.

**Figure 3:**
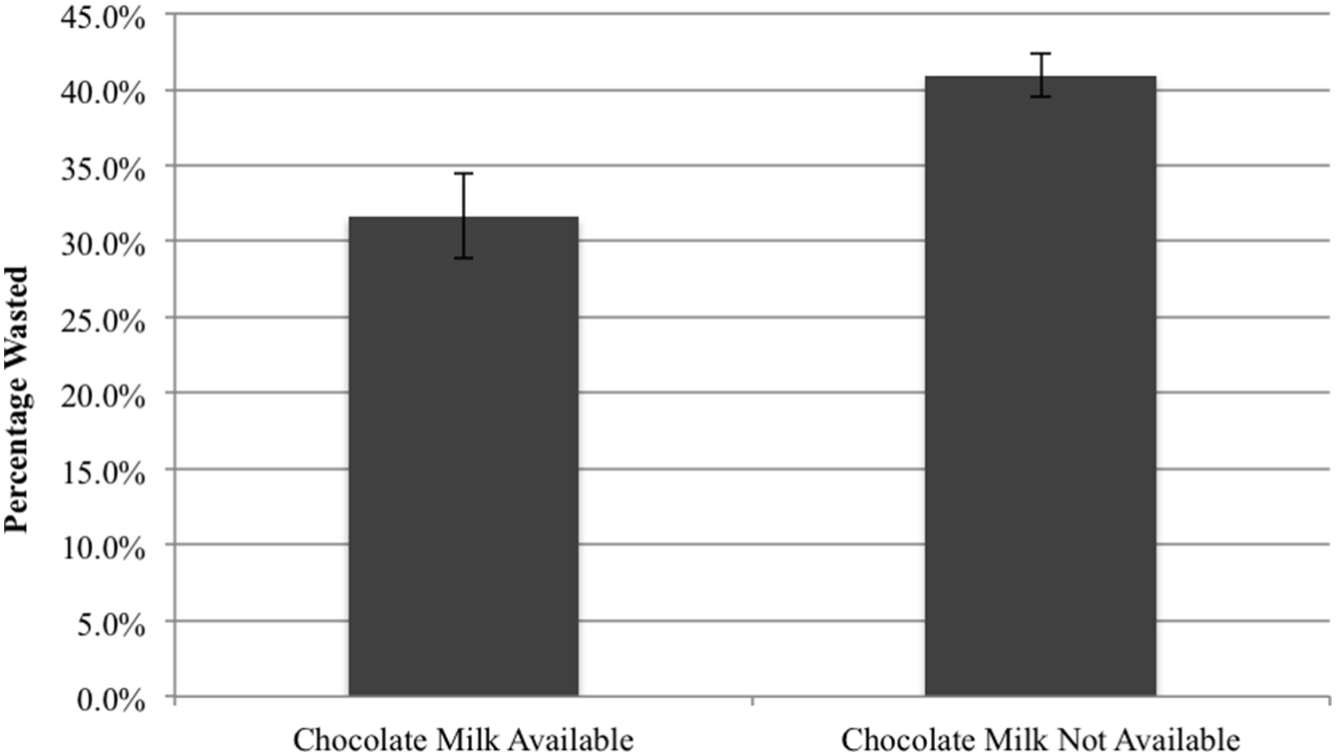
When Only White Milk Was Offered, Students Wasted More Milk.

Since milk waste was not collected during the school year when chocolate milk was still available, milk waste data in 5 elementary schools located in New York City were used as a comparison. In these schools, chocolate milk was still available. These elementary school students wasted an average of 31.7% of the milk they took. This suggests that eliminating chocolate milk can increase total milk waste by 29.4% (p<0.001; Figure 3). Based on this finding, for each additional carton of white milk taken, an additional $0.02 was thrown away, increasing the cost of white milk per ounce consumed by 10.0%. Milk waste collected from a broader set of schools in the fall of 2012 indicate that 30.0% of all milk was wasted in these schools. This provides supportive evidence that the 31.7% milk waste from the schools in New York State is a valid, and perhaps a conservative measurement.

Finally, Table 3 provides enrollment and lunch sales information for the schools in the study. Between the 2010–2011 and 2011–2012 school years, average enrollment in the schools we studied remained stable (381.3 to 379.7 students – Table 1). Notably, however, there was a 6.8% decline (p<0.05) in NSLP participation between the two years (Table 1). While it is plausible that this decline can be attributable to eliminating chocolate milk, other contemporaneous confounds inhibit our ability to identify one specific cause. Still, a powerful insight, consistent with reactance theory, is that eliminating chocolate milk could decrease the number of students ordering lunches by 6.8%.

**Table 3:** Participation in the National School Lunch Program Participation Declined When Chocolate Milk Was Banned (standard errors in parentheses).

	Chocolate Milk Available(SY 2010–2011)	Chocolate Milk Not Available(SY 2011–2012)	Difference	% Difference
Average Student Enrollment	381.3	379.7	−1.5	−0.4%
(N = 22)			(4.61)	
Average Daily Participation in the National School Lunch Program	217.7	202.8	−14.9*	−6.8%
(N = 22)			(4.17)	
Free/Reduced Price Lunches	116.5	113.9	−2.6	−2.3%
(N = 22)			(3.68)	
% Enrolled Students Receiving National School Lunch Program Meal	57.1%	53.9%	−3.20%	−5.6%
(N = 22)			(0.01)	
% Free/Reduced Price Lunches	49.2%	50.5%	1.3%	2.6%
(N = 22)			(0.01)	

School level measures aggregate measures daily averages for each school. Paired t-tests were used to compare enrollment and participation between years.

*p<0.05.

**p<0.01.

***p<0.001.

## Discussion

This natural experiment was afforded by eleven elementary schools, all of which made a clean transition from offering chocolate milk to only white milk. Although the results are limited by the absence of control schools and may not be generalizable in magnitude to middle schools and high schools, these results offer important preliminary insights related to possible economic consequences of eliminating chocolate milk. Among these schools, eliminating chocolate milk was associated with a 9.9% decrease in average daily milk sales, a 10.0% increase in the cost of milk consumption, and a 29.4% increase in milk waste. Although eliminating chocolate milk can reduce the amount of sugar and calories available in a student’s lunch, it may also have unintended negative consequences to consider.

As a pilot study, these findings are not without limitations. First, other changes to the school district introduced potential confounds to the analysis, yet the mixed regression design utilizes a within school and between year analysis to reduce some of this error. In addition, it is not possible to identify the individual impact of removing chocolate milk on milk sales or milk waste without unrealistically assuming that all other effects are either insignificant or cancel each other out. Still, due to the strong preferences for chocolate milk in schools, it may be fair to assume that student beverage selection was minimally influenced by the other menu changes that occurred in the district. So while the 6.8% decrease in lunch sales is possibly attributable to eliminating chocolate milk, and is consistent with the theory of reactance (resisting threats to freedom), the decline could also be associated with other factors. [12], [13] Identifying the actual magnitude of this phenomenon is a promising avenue for future research.

This exploratory study underscores the need for full-scale follow-up studies. In order to more accurately document the increase in waste, before and after measures should be collected. Additionally, the data for this study were collected in elementary schools only, were collected for two months, and were of an aggregate nature. Without specific age categories, and additional grade levels (6–12) the results are limited to a small age group. Furthermore, all relationships are correlational because the design is not a randomized controlled design. Moreover, we were not able to divide milk sales into units selected by students taking an NSLP qualifying meal and units purchased by other students. Thus, a properly designed experiment that collects information across ages and for NSLP and non-NSLP participants would be an appropriate follow-up study.

While this exploratory analysis examined the economic-related considerations of eliminating chocolate milk, it is also important that future research explore some of the less obvious nutrition-related trade-offs that might occur. In addition to examining potential changes in protein, calories, and calcium, it would be important to document whether students compensated at lunch, or after school, by consuming higher calorie beverages or other calorically dense snacks. Though our data did not allow us to determine whether students compensated for calories and nutrients when chocolate milk was not available, our estimates of increased milk waste and decline in cartons selected provide evidence that students were less satisfied with the set of milk options. Moreover, research suggests that small indulgences can reduce the chances for within-meal calorie compensation. [10] Given this evidence, there is need for additional research to understand how behavioral nudges and triggers that preserve options, such as chocolate milk, and guide students to more healthful choices [17], [22], [23], [24] could lead students to take, and eat, healthier lunches. After all, it’s not nutrition until it’s eaten.

## Conclusion

While removing chocolate milk from school cafeterias may appear to have the immediate benefit of reducing calorie and sugar consumption, there might be unexpected consequences to doing so. Our results indicate that when chocolate milk was removed, fewer students took milk, and students wasted more of the white milk they selected. In addition, there could also be consequences to how students compensate during lunch–or later in the day–such as selecting a dessert. Food service managers need to carefully weigh the costs and benefits of eliminating chocolate milk and should consider other solutions, such as making chocolate milk less convenient to select and making white milk appear more convenient, attractive, and normal. [25] While there are many ways to promote white milk selection without restricting available options, the following five suggestions are consistent with previous research conducted in school lunchrooms: 1) keeping all beverage coolers stocked with at least some white milk [23]; 2) white milk representing 1/3 or more of all visible milk in the lunchroom [25]; 3) placing white milk in front of other beverages, including chocolate milk, in all coolers [26]; 4) placing white milk crates so that they are the first beverage option seen in all milk coolers [22], [27]; and 5) bundling white milk with all grab and go meals available to students as the default beverage [24].

Encouraging students to take white milk can naturally decrease the amount of chocolate milk taken. While making white milk relatively more convenient, attractive, and normal to choose (relative to chocolate milk) will lead some children to switch from chocolate to white, it will not influence all children. Yet, the remaining question is whether or not drinking chocolate milk is better than drinking an alternative caloric drink, such as a sports drink, or even not eating a school lunch at all.
